# Heavy metals bioaccumulation and histopathological changes in *Auchenoglanis occidentalis* fish from Tiga dam, Nigeria

**DOI:** 10.1186/s40201-015-0222-y

**Published:** 2015-10-06

**Authors:** Samson Eneojo Abalaka

**Affiliations:** Department of Veterinary Pathology, University of Abuja, Abuja, Nigeria

**Keywords:** *Auchenoglanis occidentalis*, Heavy metals, Bioaccumulation, Histopathology

## Abstract

The assessment of heavy metals concentration in Tiga dam, Nigeria vis-à-vis their bioaccumulation and histopathological alterations in *Auchenoglanis occidentalis* from it were carried out. The water of the dam and the liver of the fish were analyzed for zinc (Zn), cadmium (Cd), lead (Pb) and iron (Fe) concentrations and their bioaccumulation factors. At the same time, the gills, liver and kidney of the exposed fish were also examined for histopathological alterations. The results revealed that concentrations of the metals differs significantly (p < 0.05) between the dam’s water and the liver of the sampled fish. Liver bioaccumulations of the metals were in the order of Zn > Fe > Cd > Pb. However, the degree of tissue alterations in the gills showed their normal functioning despite the observed alterations while liver and kidney were mildly and moderately damaged, respectively. This indicated that Zn, Cd, Pb and Fe polluted the dam.

## Introduction

Pollution of aquatic environment is a serious and growing problem [1], which is usually brought about by increasing domestic, agricultural, commercial and industrial activities of man [2]. However, heavy metals are considered the most hazardous of all environmental pollutants [3] due to their bioaccumulation and toxicity tendency [4]. This is because heavy metals may precipitate, get absorbed on sediment particles, remain soluble or suspended in water and/or may be taken up by aquatic fauna upon their entry into water bodies [5, 6]. Metals are then absorbed through gills and skin and/or ingested through food to cause bioaccumulative toxicity in fish where the intensity of the toxicity is influenced by the temperature, oxygen concentration, pH and hardness of the water [7]. Tiga dam is a huge water reservoir on Kano River in northern part of Nigeria, which is an important source of fish and water for drinking and irrigation of surrounding farm lands.

Though it derives its water source from the Jos highlands, which is known for its mining activities, it flows towards the Hadejia-Nguru wetlands through Kano to empty into Lake Chad. Therefore, there is a need to assess the concentrations and bioaccumulation status of some heavy metals and their toxic effects in fish from this big aquatic ecosystem because of the large areas it traverses. The presence of some heavy metals has been reported in Tiga dam, Nigeria [8]. Similarly, some heavy metals have also been reported in the bones, muscles and gills of Tilapia and *Clarias lazera* inhabiting the dam [9]. However, there is currently no report on the assessment of heavy metals bioaccumulation in Tiga dam and their pathological implications in the exposed fish. *Auchenoglanis occidentalis*, which is of commercial importance in Nigeria, is one of the fish that are normally caught from Tiga dam for human consumption. That is why this study aimed to conduct the assessment of heavy metals concentration in Tiga dam, Nigeria vis-à-vis their bioaccumulation and pathological alterations in *A. occidentalis* harvested from it.

## Materials and methods

### Study area

**Fig. 1:**
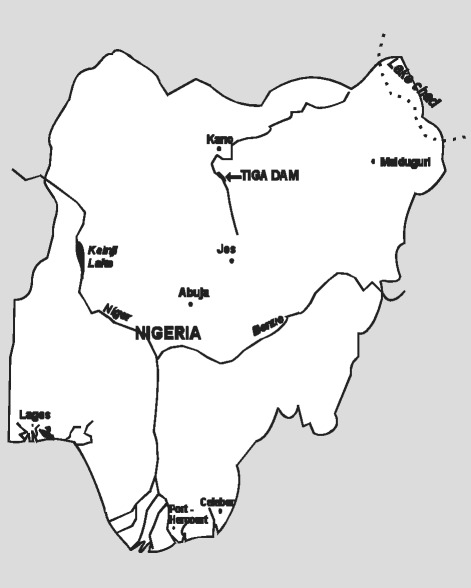
The map of Nigeria showing Tiga dam

Tiga dam is situated in Kano State, which is in northern part of Nigeria as shown in Fig. 1. It is located at Latitude 11° 15 to 11° 29″N and Longitude 8° 16 to 8° 38″E. The dam measures about 40.40 km in length, 24.40 km in width and 40.00 m in depth with a surface area of 178.00 km^2^, including a storage capacity of about 1978.49 × 10^6^ m^3^ litres of water [10], respectively.

### Water and fish sampling

A total of 13 water samples were collected once a week from different locations in Tiga dam over a 4-month period between September and December, 2009. Similarly, a total of 16 *A. occidentalis* of 435.60 ± 24.13 g mean weight and 31.41 ± 2.28 cm mean length were collected once a week from the dam by local fishermen using dug-out canoes and nets over the same period. The fish were kept in containers with fresh water from Tiga dam, Nigeria immediately upon their capture and then sampled within three hours as suggested by [11].

### Heavy metal analysis

The concentration of cadmium (Cd), lead (Pb), zinc (Zn) and iron (Fe) in the water and in the liver of the sampled *A. occidentalis* were determined using Atomic absorbance spectrophotometer (AA, 6800) based on Beer-Lambert’s law. The liver was initially digested prior to metal analysis. One gramme of the harvested liver tissue was put into a crucible and dry ashed in a furnace at 550 °C for one hour [12] before the addition of 5 ml of concentrated nitric acid and 15 ml of concentrated hydrochloric acid (1:3 v/v) in an Aqua regia digestion [13]. The mixture was then heated at 100 °C to about dryness on a hot plate before adding 10 ml of distilled water and filtered warm into a 100 ml volumetric flask, which was later made up to the mark with distilled water [14]. The absorption wavelength used for the measurements and detection limits of the metals are as shown in Table 1.

**Table 1:** The absorption wavelength and detection limit of zinc, cadmium, lead and iron under investigation

Metal	Symbol	Absorbance wavelength (nm)	Detection limit (ppm)
Zinc	Zn	213.9	-
Cadmium	Cd	228.8	0.0028
Lead	Pb	217.0	0.02
Iron	Fe	248.3	0.59

### Bioaccumulation factor

Bioaccumulation factor was determined on the basis of the ratio of a particular metal concentration in any organ and its dissolved concentration in the water as described by [15].

### Histopathological examination

The fish were euthanized with 40 % ethyl alcohol before individually excising their gills, liver and kidney for histopathological processing and examinations. Tissues were fixed in 10 % formal saline prior to paraffin embedding, sectioning at 5 μm and then staining with haematoxylin and eosin [16, 17]. The severity of the lesions in each of the organ was determined semi-quantitatively by modifying the degree of tissue changes (DTC) method described by [18]. Alterations in each of the organ were classified in progressive order based on the formula: DTC = (1 × Σ *I*) + (10 × Σ *II*) + (100 × Σ *III*) after screening the number of tissue lesions in stages *I*, *II* and *III* for that particular organ. Alterations that do not alter the normal functioning of the organ were classified as Stage *I* alterations. Those alterations that were more severe and impaired the normal functioning of the organ were classified as stage *II* alterations. Similarly, severe alterations that induce irreparable tissue damage were classified as stage *III* alterations. Organs with numerical values ranging from 0 – 10 were graded as normal tissue, 11 – 20 as mildly damaged tissue, 21 – 50 as moderately damaged tissue, 51 – 100 as severely damaged tissue while those with numerical values above 100 were graded as irreversibly damaged tissue.

### Statistical analysis

GraphPad software programme (GraphPad Prism, version 4.0, San Diego, California, USA. www.graphpad.com) was used to determine the mean (± sem), which were further subjected to one-way analysis of variance (ANOVA), Student’s *t*-test and Tukey’s tests for comparing differences between the means for statistical significance (*p* < 0.05).

## Results and discussion

The mean concentrations of Pb, Cd, Zn and Fe in the dam water as well as in the liver of the sampled *A. occidentalis* and their bioaccumulation factors were determined as shown in Table 2. The concentrations of Pb, Cd, Zn and Fe were higher in the liver of *A. occidentalis* than their concentrations in the water of the dam in affirmation of an earlier report by [19] that lower concentrations of metals are generally recorded in water compared to fish tissues. These might be attributed to the pollution of the fish’s external environment and the rate of ingestion and excretion in the exposed fish [20]. The significant (*P* < 0.05) differences between the concentrations of the metals in the dam’s water and also in the liver of *A. occidentalis* might be due to varying levels of the pollution of the dam with the metals. Lead might have come from sewage and agricultural wastes discharged into the dam [4]. Similarly, Cd might have come from natural sources and/or as run-off from agricultural soils around the dam where phosphate fertilizers have been in use [21, 22]. However, Zn might have arisen from the mining activities within the Jos plateau, which is the main source of the dam’s water along with run-offs from around it. Although lead was the least bioaccumulated metal, its water and liver concentrations were higher than the recommended maximum acceptable limits of 0.01 mgkg^-1^for water and 0.01 mgkg^-1^ for fish tissues [23, 24]. Similarly, the concentrations of Cd and Fe in Tiga dam, Nigeria and in the liver of the fish were also above the maximum acceptable limits recommended by [23, 24] for both water and fish tissues, respectively. High concentrations of Pb and Cd in both the dam’s water and in the liver of the fish tended towards toxic concentrations. This is because although Pb and Cd are non-essential for metabolic activity, they exhibit toxic properties even at trace concentrations [25]. The toxic effect of Pb and Cd might be in the form of severe membrane integrity damage with subsequent loss of membrane-bound enzyme activity resulting in cellular death [26]. Iron is found in natural freshwaters and has no health guideline value but high concentration in water may give rise to consumer complaints [27]. Although zinc was the most bioaccumulated metal, its water and liver concentrations were below the recommended maximum acceptable limits [23, 24]. However, zinc and iron are essential metals for the growth and well-being of organisms but they may bioaccumulate beyond the optimum threshold concentrations to becoming hazardous and toxic [5]. This is because zinc is reported to be potentially toxic to fish [28] and may cause structural damage in the exposed fish [29].

**Table 2:** Mean concentrations of lead, cadmium, zinc and iron in the water and liver of *Auchenoglanis occidentalis* sampled from Tiga dam, Nigeria (*n* = 13 for water; *n* = 16 for fish)

Parameters	Lead (Pb)	Cadmium (Cd)	Zinc (Zn)	Iron (Fe)	References
Water concentration (ppm)	0.33 ± 0.03	0.02 ± 0.00*	0.01 ± 0.00**	2.61 ± 0.07**	This study
Liver concentration (ppm)	0.35 ± 0.04	0.04 ± 0.00*	2.17 ± 0.30**	11.66 ± 1.32**	This study
Bioaccumulation factor	1.06	2.00	217.00	4.46	This study
Maximum acceptable water limit (ppm)	0.01	0.003	3.00	0.30	NIS (2007)
	0.01	0.01	5.00	0.30	WHO (2003) [24]
Maximum acceptable liver limit (mgkg^-1^)	0.01	0.01	5.00	0.30	WHO (2003) [24]

*:Values within the column are statistically significant at *p* < 0.0056

**:Values within the column are statistically significant at *p* < 0.0001

*NIS* Nigerian Industrial Standard

*WHO* World Health Organization

**Fig. 2:**
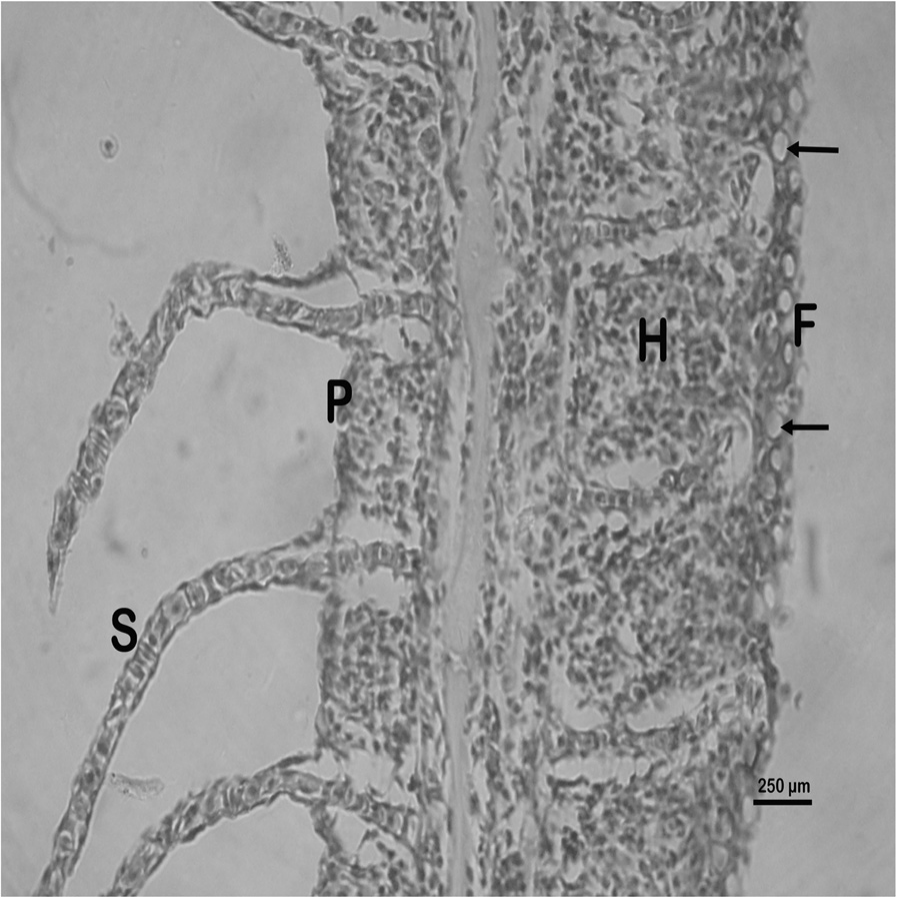
Photomicrograph of the gill of *Auchenoglanis occidentalis* sampled from Tiga dam, Nigeria. Note the primary lamellar (P), secondary lamellar (S), epithelial hyperplasia (H), complete lamellae fusion (F) and mucous cells hyperplasia and hypertrophy (arrows)

**Fig. 3:**
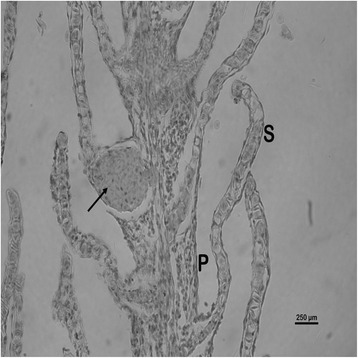
Photomicrograph of the gill of *Auchenoglanis occidentalis* sampled from Tiga dam, Nigeria. Note the primary lamellar (P), secondary lamellar (S) and aneurysm (arrow)

**Fig. 4:**
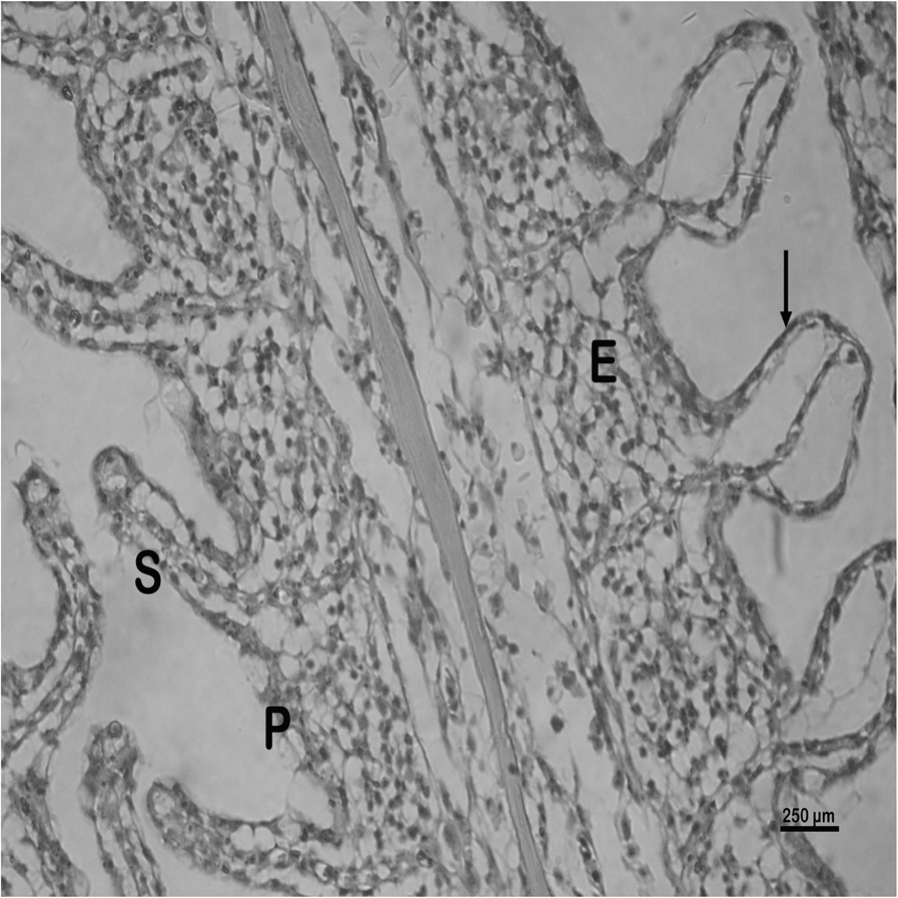
Photomicrograph of the gill of *Auchenoglanis occidentalis* sampled from Tiga dam, Nigeria. Note the primary lamellar (P), secondary lamellar (S), lamellae oedema (E) and epithelial detachment (arrow)

**Fig. 5:**
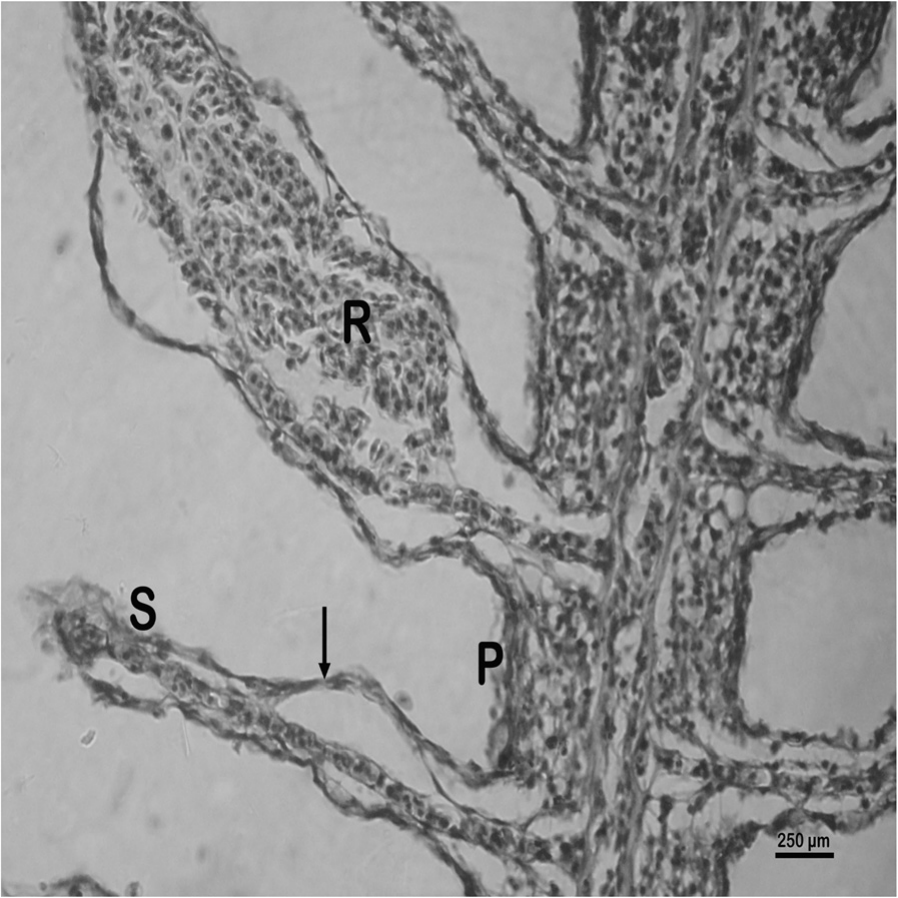
Photomicrograph of the gill of *Auchenoglanis occidentalis* sampled from Tiga dam, Nigeria. Note the primary lamellar (P), secondary lamellar (S), haemorrhage (R) and epithelial detachment (arrows)

**Fig. 6:**
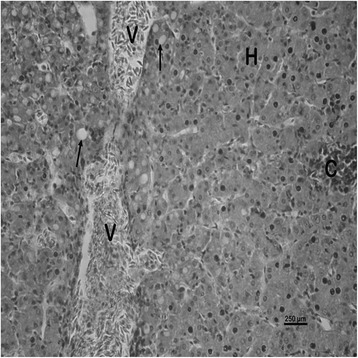
Photomicrograph of the liver of *Auchenoglanis occidentalis* sampled from Tiga dam, Nigeria. Note the hepatocyte (H), central vein (V), cellular infiltration (C) and vacuolations (arrows)

**Fig. 7:**
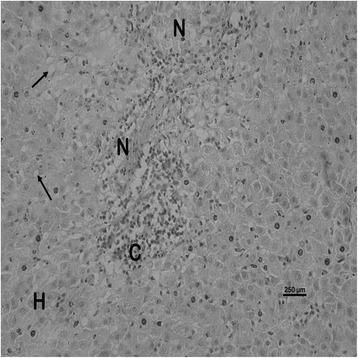
Photomicrograph of the liver of *Auchenoglanis occidentalis* sampled from Tiga dam, Nigeria. Note the hepatocyte (H), cellular infiltration (C), necrosis (N) and vacuolations (arrows)

**Fig. 8:**
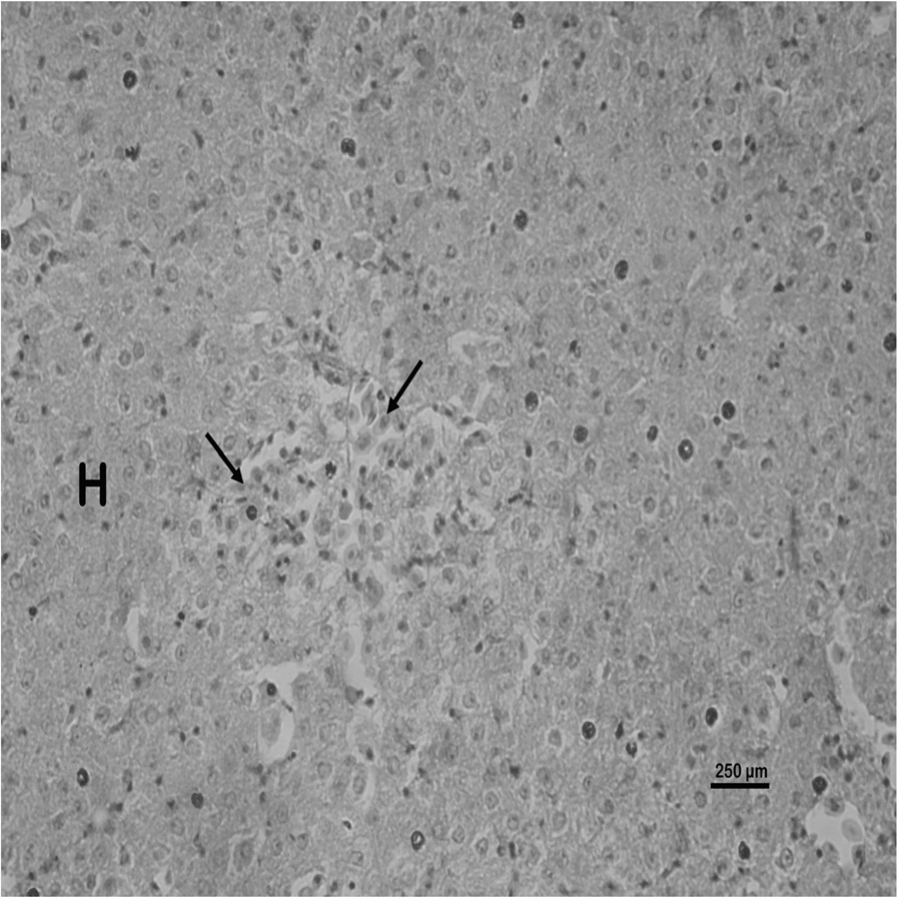
Photomicrograph of the liver of *Auchenoglanis occidentalis* sampled from Tiga dam, Nigeria. Note the hepatocyte (H) and haemorrhage (arrows)

**Fig. 9:**
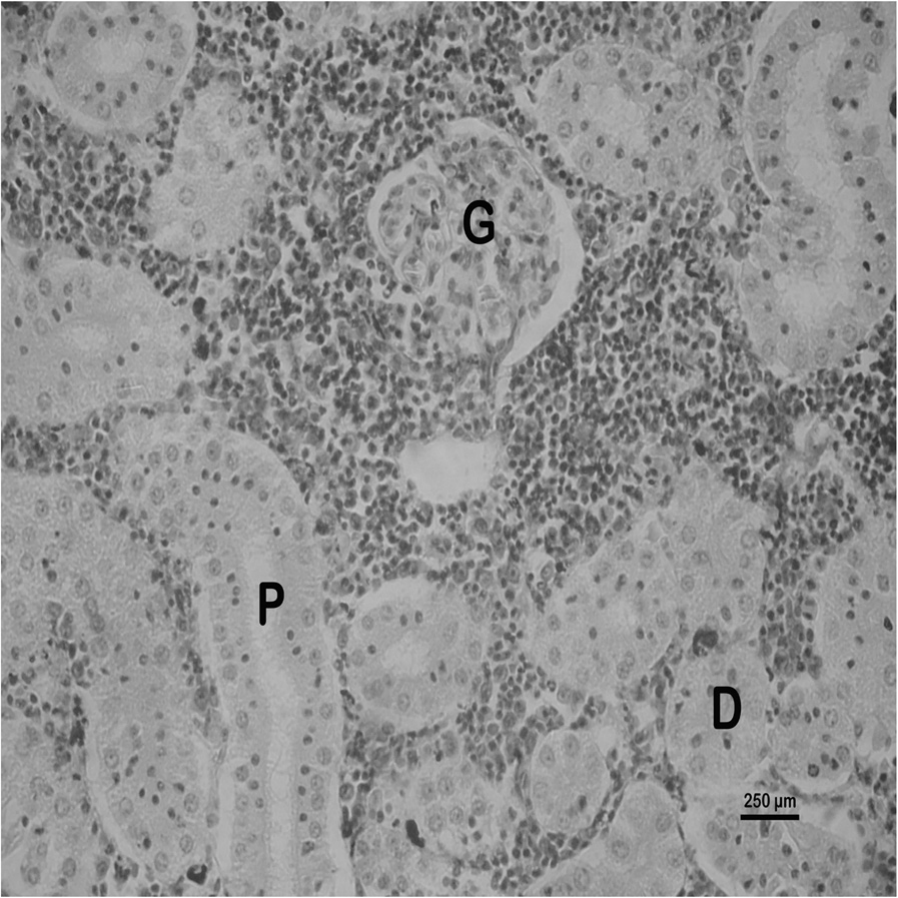
Photomicrograph of the kidney of *Auchenoglanis occidentalis* sampled from Tiga dam, Nigeria. Note the glomerulus (G), proximal tubule (P) and the distal tubule (D)

**Fig. 10:**
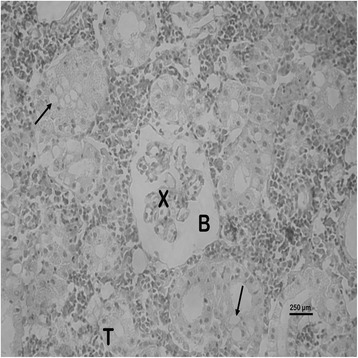
Photomicrograph of the kidney of *Auchenoglanis occidentalis* sampled from Tiga dam, Nigeria. Note glomerular necrosis (X), tubular necrosis (T), widened Bowman’s space (B) and tubular vacuolations (arrows)

**Fig. 11:**
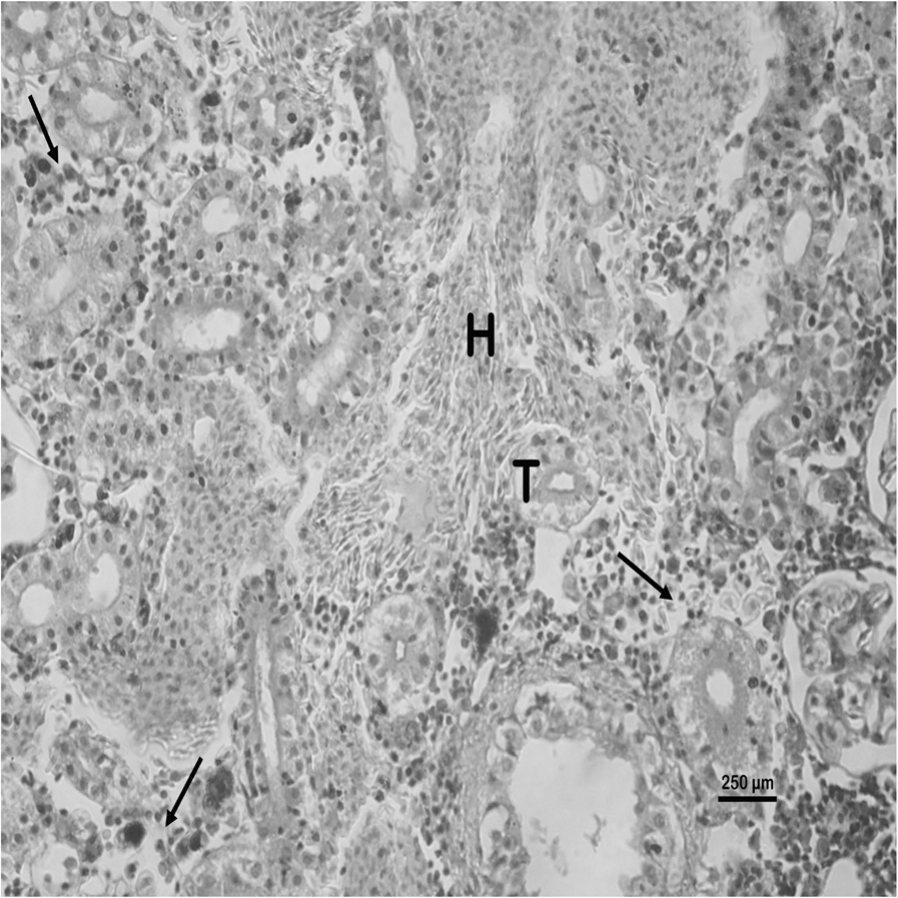
Photomicrograph of the kidney of *Auchenoglanis occidentalis* sampled from Tiga dam, Nigeria. Note haemorrhage (H), tubular necrosis (T) and interstitial necrosis (arrows)

The nature and severity of the observed lesions in the gills, liver and kidney of the sampled fish are as shown in Table 3 while the observed histopathological alterations in the gills, liver and kidney are also presented in Figs. 2, 3, 4, 5, 6, 7, 8, 9, 10, 11, respectively. The degree of tissue change (DTC) in the gill of the sampled fish was calculated to be 9.75 ± 3.00 while that in the liver of the fish was 14.06 ± 9.46. Similarly, the DTC in the kidney of the sampled fish was 26.63 ± 11.16. However, there were no significant (p > 0.05) differences in the DTC between the gills, liver and kidney of exposed fish. The observed epithelial proliferation (hyperplasia) and the detachment of the gill epithelial cells along with the partial to complete lamellar fusion are non-specific responses of the gills to toxic irritants [30], which were natural attempts by the exposed fish to increase the diffusion distance between their blood and the toxic external environment [31–33]. Increased permeability of the gill capillary walls following vessel dilatation at the site of toxic damage might be responsible for the observed lamellar oedema [34]. The observed mucous cells proliferation and hypertrophy, which leads to increased mucous secretion, were protective defence responses to coat absorptive surfaces [30] in line with the fact that increased mucous secretions are more often associated with heavy metal exposure than with organic pollutant exposures [30].

**Table 3:** Histopathological lesions in the gills, liver and kidney of *Auchenoglanis occidentalis* sampled from Tiga dam, Nigeria

Stage	Degree of tissue change		
	Gills	Liver	Kidney
	Alteration %	Alteration %	Alteration %
*I*	Epithelial proliferation 50.0	Vacuolation 12.5	Vacuolation 18.8
	Epithelial detachment 43.8	Cellular infiltration 18.8	Widened bowman's capsule 12.5
	Lamellar oedema 56.3		
	Mucous cells proliferation 12.5		
	Mucous cells hypertrophy 12.5		
	Partial lamellar fusion 50.0		
*II*	Complete lamellar fusion 18.8	Haemorrhage 12.5	Haemorrhage 12.5
	Aneurysm 43.8		
	Haemorrhage 12.5		
*III*		Hepatic necrosis 12.5	Glomerular necrosis 6.3
			Tubular necrosis 6.3
			Interstitial necrosis 12.5

The observed hepatic vacuolations may be due to lipid and/or glycogen deposition [35] suggestive of metabolic disorders as a consequence of the exposure to toxic agents [36]. Hepatic cellular infiltrations were suggestive of inflammatory responses in the affected liver. The observed haemorrhage might be due to the toxic damage to the liver of the exposed fish while the necrotic changes in the affected liver were a consequence of the toxic effects of the metals [6]. These hepatic lesions might probably be due to the primary function of the liver in the metabolism and excretion of toxicants where some morphological changes do occur in the process [37]. Although liver lesions are not usually specific to pollutants, a casual relationship between metal concentrations and fish liver lesions has been established [38]. This might be why the estimated DTC in the liver of the exposed fish suggested mild organ damage. The choice of the gills and the liver as monitor tissues for this study was due to their known functions in the uptake, bioaccumulation and the detoxification of environmental pollutants [39–41]. Similar changes have been reported in the gills and liver of exposed fish [42, 43].

The observed tubular vacuolations in the kidney of the sampled fish were indicative of fatty degenerative changes due to metabolic disorders. This is because cytoplasmic vacuolations are known to occur in parenchyma cells (hepatic cells, kidney tubular epithelium and myocardial cells) in cases of fatty overload [44]. The observed tubular necrosis was indicative of exposure to toxic chemical substances [45]. These changes are in agreement with the fact that heavy metals are known to cause cellular damages in the kidney of exposed fish [46, 47]. The observed histopathological lesions in the gills, liver and kidney of the exposed *A. occidentalis* were similar to lesions reported in same organs of *Oreochromis niloticus* exposed to some heavy metals [48].

## Conclusion

The increased water concentrations of Pb, Fe and Cd far above the maximum acceptable limit and their bioaccumulation in the liver of the sampled *A. occidentalis* were suggestive of heavy metals pollution. This is further reinforced by the observed alterations in the gills, liver and kidney of the sampled *A. occidentalis*. This revelation call for great concerns and highlight the need for constant monitoring of this huge and important water body in order to safeguard the health and lives of organisms, animals and people associated with it.
